# Case report: Low-dose radiation-induced meningioma with a short latency period

**DOI:** 10.3389/fonc.2024.1413610

**Published:** 2024-07-01

**Authors:** Jinyang Li, Xiangmao Zhang, Jing Liu, Chunxia Su, Junxiang Cui, Liling Yang, Yinghao Gu

**Affiliations:** ^1^ School of Clinical Medicine, Shandong Second Medical University, Weifang, China; ^2^ Department of Neurosurgery, Zibo Central Hospital, Zibo, China; ^3^ Department of Pharmacy Intravenous Admixture Service, Zibo Central Hospital, Zibo, China; ^4^ Department of Admissions Service Center, Zibo Central Hospital, Zibo, China

**Keywords:** meningioma, radiation-induced meningioma, RIM, radiotherapy, ionizing radiation, oncology

## Abstract

Patients with radiation-induced meningioma (RIM), most of whom had received head radiation therapy or had been exposed to ionizing radiation during childhood or adolescence, are at risk of developing cranial meningiomas throughout their lifetimes because of the long latency period. Although intermediate-to-high–dose ionizing radiation exposure is an established risk factor for RIM, risk factors for low-dose RIM remain incompletely defined. This study presents the case of a 56-year-old woman diagnosed with radiation-induced giant meningioma 2.5 years after undergoing an interventional embolization procedure for a brain aneurysm. This is the first report of RIM attributable to a brain intervention with an extremely short latency period. The total radiation dose received by the patient during the operation was 1367.3 mGy, representing a low dose. Our case report strengthens the evidence that even low radiation doses can increase the risk of RIM. These findings provide a realistic basis for the theoretical study of RIM and suggest some new ideas for RIM treatment. The need for caution in the use of radioactive treatments and optimization of interventional procedures is highlighted.

## Introduction

The use of radiologic therapeutic measures such as computed tomography (CT), X-rays, radiotherapy, angiography, and interventional procedures in clinical practice has rapidly increased with technological advances. Although the benefits of these techniques in patient management are unquestionable, the cumulative dose of radiation over a long period or with frequent application can increase the risk of cancer (1–3). Meningiomas are mostly benign tumors originating from arachnoid cap cells. They are the most common benign intracranial tumors, accounting for 13%–26% of all primary intracranial tumors (4–6). Radiation-induced meningioma (RIM) is the most common brain tumor known to be caused by ionizing radiation (7, 8). In 1933, Lacassagne first proposed this concept in an animal model, and in 1953, Mann et al. reported the first case of RIM (9). Later, Cahan established the diagnostic criteria for RIM (10), and Harrison categorized RIM as low-, medium-, and high-dose lesions according to the cumulative dose of radiation (11). Current research on RIM focuses on etiology, epidemiology, and prognostic factors. In this study, we present the case of a 56-year-old woman diagnosed with low-dose RIM only 2.5 years after an interventional surgery. The patient’s symptoms were linguistic confusion, left limb immobility, and drowsiness. Emergency CT and magnetic resonance imaging (MRI) revealed an intracranial lesion, and the patient’s condition deteriorated during the examination. Immediate surgery was performed to remove the intracranial tumor. To the best of our knowledge, this is the first reported case of RIM triggered by a brain interventional procedure. Our case provides new evidence for the risk factors of low-dose RIM, and by reviewing the related literature, our report also provides a realistic basis for some theoretical studies of RIM and suggests some new ideas for its management. In the future, the sample size should be expanded to thoroughly study the influence of the radiation dose in brain interventional procedures, and the findings should be used as a basis to optimize such procedures and reduce the risk of low-dose RIM.

## Case presentation

**Figure 1:**
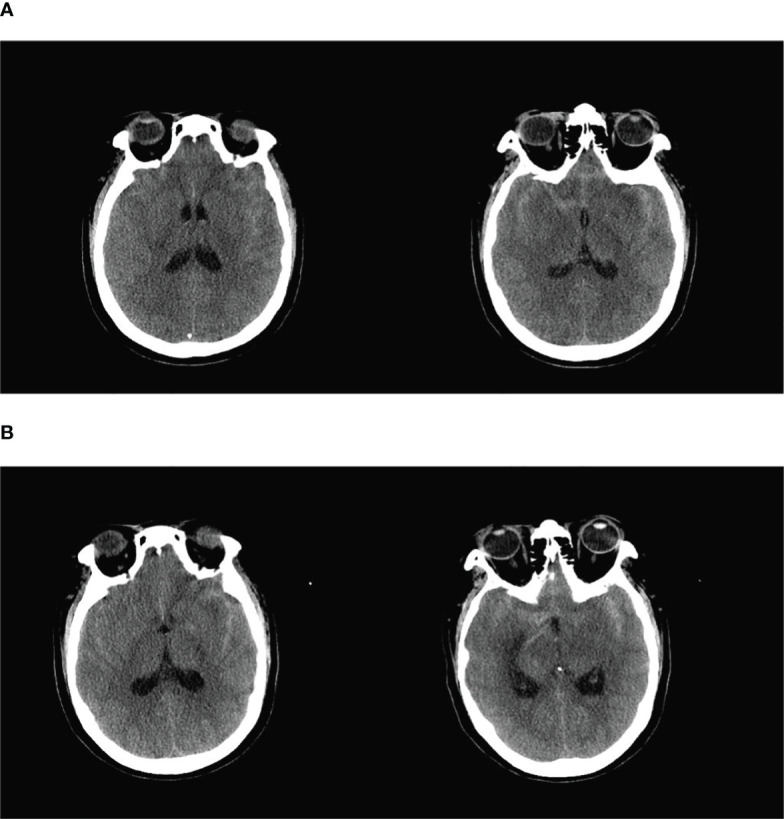
**(A)** The basal pools, ring pools, tetrapodal pools, and lateral fissure pools were all visible as hyperdense shadows with a full brain parenchyma and flattened sulcus gyrus. The initial diagnoses were subarachnoid hemorrhage and brain swelling. **(B)** Dense shadows were present in the bilateral lateral fissure pools and in part of the left parietal sulcus, and the hemorrhagic portion had decreased in size.

**Figure 2:**
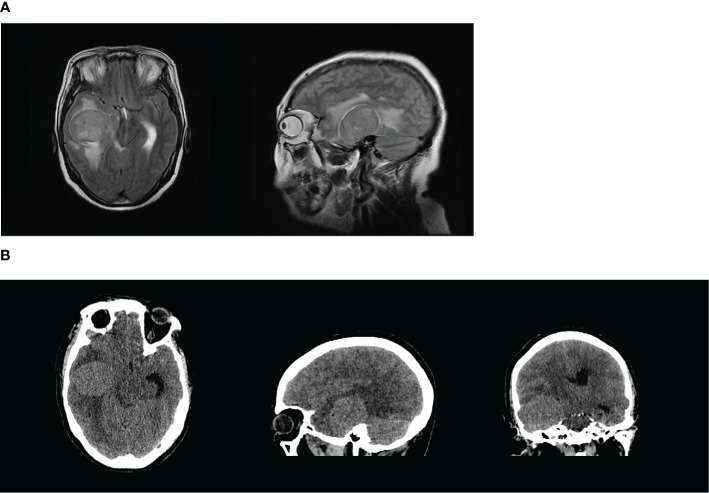
**(A, B)** The right temporal lobe had an abnormal signal shadow similar to a circle. The shadow measured approximately 5.1 × 4.1 cm^2^ with a clear border, and the adjacent lateral ventricle was compressed and deformed, with a leftward deviation of the midline of approximately 0.5 cm. These findings were consistent with the manifestation of a tumor, and the lesion was considered more likely to be a meningioma.

**Figure 3:**
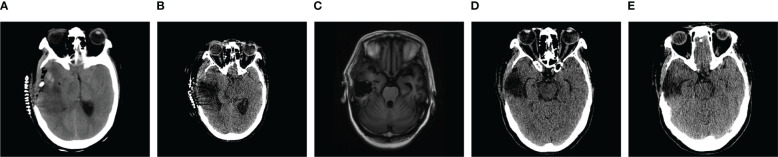
**(A)** The right side of the skull was partially missing, irregular flaky mixed density shadows were present in the right temporal lobe, and the ventricular system was narrowed by compression, with a leftward shift of the midline of approximately 0.5 cm. **(B)** The right side of the skull was partially missing, the hyperdense and isodense shadows in right temporal lobe were less dense than previously observed, the ventricular system was narrowed by compression, and the midline was shifted to the left by approximately 0.3 cm. **(C)** Partial absence of the right cranium centered in the midline. **(D)** The right side of the skull was partially missing, irregular flaky hypodense shadows were present in the right temporal lobe, and the ventricles were structurally sound and centered on the midline. **(E)** The right side of the skull was partially missing, the right temporal lobe had an irregular flaky hypodense shadow that was significantly less extensive than previously observed, and the ventricular system was fair and centered on the midline.

**Figure 4:**
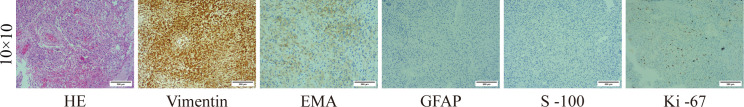
(Right temporal lobe) Meningothelial meningioma, WHO grade I. Vimentin (+), EMA: portion (+), GFAP (−), S-100 (−), Ki-67 (+).

The patient was a 56-year-old woman who underwent embolization of an intracranial aneurysm 2.5 years before presentation. The patient was admitted to the hospital on June 20, 2019 with a sudden onset of impaired consciousness, headache, dizziness, and vomiting for 4 h. Cranial CT (**Figure 1A**) revealed subarachnoid hemorrhage, which was considered a ruptured intracranial aneurysm. Total cerebral angiography and interventional embolization of the intracranial aneurysm were performed under general anesthesia. Intraoperatively, abnormal protrusion of the apical portion of the basilar artery measuring approximately 3.37 × 2.14 × 2.82 mm^3^ was found, and tight embolization was performed. According to the records, the duration of the surgery was 1.5 h. The patient received a radiation dose of 1367.3 mGy during this procedure. The patient’s postoperative course was uneventful, and the outcome was favorable. No intracranial tumor was detected on preoperative or postoperative cranial CT (**Figure 1B**). However, the patient did not follow the doctor’s orders for review until 2.5 years after the surgery. The patient was admitted to the hospital with sudden linguistic confusion, left limb immobility, and drowsiness. Urgent cranial MRI and CT revealed a right temporal lobe occupancy with significant displacement of midline structures, brainstem compression, and deformation but no dilatation of the ventricular system, and no enhanced scan was performed (**Figures 2A, B**). During the examination, the patient’s condition gradually deteriorated, leading to unconsciousness and an inability to answer questions, resulting in a Glasgow coma scale (GCS) score of 6. Additionally, her right pupil diameter was 6 mm, and her left pupil diameter was 3 mm. Bilateral pupillary direct and indirect light responses were absent. The muscle strength of the left limb was grade I, and that of the right limb was grade III. The left Babinski sign was positive. The initial diagnoses were right temporal lobe occupation and brain herniation. The patient’s critical condition necessitated emergency tumor resection via a right enlarged pterygoid approach and decompressive craniectomy under general anesthesia. Intraoperatively, a small portion of tumor tissue bulged through the bone window, but intraoperative ultrasound indicated no blood flow signal, eliminating the possibility of a large intracranial aneurysm. The tumor tissue had an intact thicker envelope after incision, clear borders defined with surrounding brain tissue, and edema of peripheral normal brain tissue. The tumor, measuring approximately 5 × 5 × 5 cm^3^ in size, was adherent to the dura mater at the base of the skull and located at the right mid-cranial base. The tumor was resected in chunks, and the adherent basal dura mater was cauterized using bipolar electrocoagulation to prevent remnants of tumor tissue. Brain tissue collapsed after tumor resection, and drains were placed in the cavity, followed by the placement of artificial dura mater and drains outside the dura mater. Because of preoperative brain herniation, the bone flap was no longer retracted, and decompression of the bone flap was performed. The skull was closed layer by layer, and the patient was admitted to the neurosurgery intensive care unit with mannitol and hormones to eliminate cerebral edema and control intracranial pressure. Based on the postoperative physical examination, the patient’s GCS score was 12. The bilateral pupils were equal in size and round but dull to light reflection. The muscle strength of the left limb was grade 3, and that of the right limb was grade 4. The patient’s postoperative CT confirmed that the tumor had been completely resected, with no significant bleeding in the operative area (**Figure 3A**). Histopathological examination confirmed that the right temporal lobe lesion was a meningioma [meningothelial, World Health Organization (WHO) grade I; **Figure 4**]. On the fifth postoperative day, the patient developed fever, lumbar puncture suggested an intracranial pressure of 210 mmH_2_O, and the color of cerebrospinal fluid was slightly yellowish and turbid. Therefore, the possibility of intracranial infection was considered, and the patient was administered meropenem + vancomycin. Physical examination illustrated that the patient’s GCS score was 12, the pupils were equal in size and sensitive to light reflexes, and muscle strength was grade 4 in the left limb and grade 5 in the right limb (**Figure 3B**). On postoperative day 23, the patient’s condition was stable, physical examination revealed a GCS score of 15, and the pupils were equal in size and sensitive to light reflex. The muscle strength of the left limb was grade 4, and that of the right limb was grade 5. MRI revealed that the meningioma had been completely resected (**Figure 3C**). The patient was discharged from the hospital waiting for review with a recommendation for MRI every 3–6 months. Considering the patient’s financial situation, we chose the less expensive CT instead of MRI at the time of the review. Follow-up CT 2 and 5 months after surgery revealed no residual or recurrent tumor (**Figures 3D, E**), and the patient was able to return to normal activities without epilepsy, visual and auditory disturbances, headaches, or other sequelae.

## Discussion

RIM is a meningioma that occurs after exposure to ionizing radiation for certain diseases. In 1933, Lacassagne developed the concept of radiation-induced tumors by demonstrating tumor formation in animal models (12–15). The earliest report of meningioma after radiation therapy was published by Mann et al. in 1953 (9). In 1998, Cahan et al. (10) established the diagnostic criteria for radiation-induced brain tumors based on the following parameters: 1) the tumor must occur within the radiation field; 2) there must be a latency period between irradiation and tumor development; 3) radiation-induced tumors have a different histological type than the previous tumor; 4) patients must not have any disease conducive to tumor development, phakomatosis, tuberous sclerosis, pigmentary dry skin disease, retinoblastoma, or neurofibromatosis; 5) the tumor must not be present before radiation therapy; and 6) the tumor must not be recurrent or metastatic. According to the radiation dose, Harrison et al. (11) grouped RIMs into three categories, namely those attributable to high-dose (>20,000 mGy), intermediate-dose (10,000–20,000 mGy), and low-dose radiation (<10,000 mGy). At present, RIM is the most common brain tumor known to be caused by ionization radiation (7). The cohort study by Bowers et al. recorded a 3-year survival rate of 95% and a 5-year survival rate of 91% for RIM, with the high-risk groups comprising female patients and children, who had an 88.2% probability of neurologic sequelae at 5 years (16). Cranial irradiation is a proven etiologic risk factor for the development of meningioma (17–19). High-dose RIM is mostly observed after radiotherapy for primary and metastatic brain tumors, and intermediate-dose RIM is mostly observed after the treatment of vascular nevi and local irradiation of superficial head and neck tumors (14). Low-dose RIM has more potential causes, and even low radiation doses of 1000–2000 mGy significantly increase the risk of secondary brain tumors and neurological tumors (20). Currently known sources include the atomic bombings of Hiroshima and Nagasaki (21–24), childhood receipt of radiotherapy for tinea capitis (20, 25–29), radiological examinations of the head and neck, and stomatological X-rays (30–34). Thus, this is the first known case in which RIM was triggered by an interventional procedure. The latency period of RIM varies widely from a minimum of 12 months (35) to a maximum of 63 years (36). The mean latency period of RIM is 22.9 ± 11.4 years (24, 37). Factors affecting the latency of RIM include the radiation dose, age, and grade of pathology. The effect of the radiation dose on the latency period remains controversial. Some studies found statistically significant differences in latency between patients receiving low- and high-dose radiotherapy, as the latency period was inversely proportional to the radiation dose (11, 37–40). Contrarily, Strojan et al. described 126 cases of RIM from the literature. From the reported data, latency was inversely related to age at the time of radiation, with the latency period being significantly shorter in patients who were exposed before the age of 17. Conversely, the analysis revealed no correlation between latency and the radiation dose (22). Retrospective studies with larger sample sizes are needed to clarify this issue. According to statistics, the average latency periods of WHO grade I, II, and III meningiomas are 24.8, 21.9, and 12.9 years, respectively (37), indicating the inverse relationship between the pathology grade and latency period. In the present case, the pathologic grade was WHO grade I, but the benign tumor had an extremely short latency period with an extremely rapid growth rate. We believe these findings add new evidence for studying the pathological behavior of RIM. RIM is clinically considered separate from spontaneous meningioma (SM). Of all RIMs, 68% are WHO grade I, 27% are WHO grade II, and 5% are WHO grade III. Among SMs, 91.5% are WHO grade I, 7.1% are WHO grade II, and 1.4% are WHO grade III (14). This comparison revealed that RIM has a greater probability of developing into high-grade meningioma, a higher probability of presenting with multiple tumors, a higher recurrence rate, and greater aggressiveness (13, 15). Shoshan et al. compared RIM and SM and found that inactivation of the *NF2* gene and deletion of chromosome 22q were less common in RIM than in SM, but the probability of a chromosome 1p deletion was 57% in RIM, versus 30% in SM (41–43). However, these studies on the cytogenetic aspects of RIM had small sample sizes, and further studies are needed. In terms of cellular dynamics, the current view is that there is no correlation among the cellular dynamics, histology, and invasive behavior of RIM, which indicates that even benign RIM can exhibit invasiveness (15). The RIM we reported had WHO grade I pathology, and it was benign meningiomas. However, they grew rapidly, and clinical symptoms developed within a short latency period, highlighting a more aggressive nature than observed for ordinary meningiomas. We believe these characteristics support the cytokinetic theory of the study. Regarding the management of patients with RIM, surgical resection remains the treatment of choice (15, 44), and given the high recurrence and growth rates of RIM (40), dura and peridural bone tissue should be removed as widely as possible at the time of surgery (11). High-dose RIM should consider scalp atrophy in the radiation area, and surgical incisions can increase the potential for poor prognoses (13). The use of radiotherapy for RIM is controversial. RIMs can sometimes be treated with radiation, whereas Mathiesen found that radiotherapy is of little significance in the control of RIM (45). However, Umansky et al. concluded that the use of radiotherapy is desirable for WHO grade II–IV RIM and for lesions that cannot be radically resected (13). By reviewing the literature and considering our experience of this case, we believe that radiotherapy should be avoided after WHO grade I RIM has been radically resected, but radiotherapy can be used to reduce the risk of recurrence and prolong the time to recurrence for incompletely resectable and high-grade RIM. For RIM prevention, patients who have received radiotherapy and radiological examinations of the head should be monitored for a long period, and imaging should be performed every 3–6 months (46). Asymptomatic meningiomas can be detected by long-term testing of the patient (47–50), and such patients should be treated as early as possible to reduce the morbidity and mortality of the neurological sequelae of RIM (16, 51). In addition, we believe that non-radiological examinations such as MRI should be used in long-term monitoring to avoid further radiation exposure as a risk factor for RIM. In the current case, meningioma occurred within the irradiated area with a sufficient latency period between irradiation and meningioma development (2.5 years), and the patient had no history of a tumor or a condition conducive to tumor development prior to the diagnosis of meningioma. According to the records, the radiation dose received by the patient during this intervention was 1367.3 mGy. Eventually, we diagnosed the lesion as low-dose RIM. The meningioma in this case grew to 5 × 5 × 5 cm^3^ in approximately 2.5 years. To our knowledge, this is the first reported case of RIM triggered by a brain interventional procedure. This case adds new evidence to the risk factors for low-dose RIM. We have reviewed the literature to guide the treatment process, and in this manner, we have identified new ideas for the prevention, diagnosis, and postoperative management of RIM, which add to the current diagnostic and treatment strategies for RIM. In the course of reviewing the literature, it was found that this case provides a realistic basis for theoretical studies of the cellular dynamics of RIM. However, several limitations must be noted. First, for the postoperative review of RIM, we recommend non-radiological auxiliary examinations such as MRI, but because of the patient’s financial status, CT was selected as a less expensive alternative. Second, because the patient did not follow the medical advice for regular review after the intervention, we could not confirm the exact latency period of RIM. Although the time from the intervention to re-admission for intracranial occupancy was 2.5 years, the actual latency period might have been shorter. As a future direction of RIM research, we should expand the sample size, thoroughly study the influence of the radiation dose in brain interventional procedures, and use these findings as a basis to optimize brain interventional procedures and reduce the risk of low-dose RIM.

## Conclusion

RIM is a definable clinical entity. The most susceptible population is young children and women. Even low doses of radiation can significantly increase the incidence of RIM. A younger age at the time of initial radiation exposure and a higher pathologic grade of RIM are associated with shorter latency periods for RIM. The effect of the radiation dose on the latency period to tumor development is uncertain. RIM is more aggressive than common meningiomas, and it has a higher rate of postoperative recurrence. Surgery remains the treatment of choice for RIM, and complete resection can significantly reduce the postoperative recurrence rate. Long-term surveillance of people at high risk for RIM can detect early asymptomatic RIM, and early detection and treatment can significantly improve the prognosis of RIM. In this study, we reported a rare case of low-dose RIM occurring 2.5 years after embolization of an intracranial aneurysm. To our knowledge, this is the first patient in whom RIM was triggered by an interventional procedure, adding a new risk factor for low-dose RIM. The patient’s tumor specimen was pathologically classified as a WHO grade I benign tumor, but it had a very short latency period and exhibited a more aggressive nature than ordinary meningiomas. These characteristics provided a realistic basis for the theoretical study of RIM cell dynamics by AL-MEFTY et al. Based on the treatment experience of this case, we suggest that RIM generally has a long latency period, and people at high risk of RIM, including those undergoing cerebral angiography and cerebral vascular interventions, should undergo lifelong monitoring using non-radiological auxiliary examinations such as MRI. Second, considering the excellent prognosis of total surgical resection and the specificity of RIM triggers, postoperative radiotherapy should be avoided in WHO grade I RIM. We believe that this will provide a new reference for the diagnosis and treatment of RIM. Considering the high sensitivity of meningeal tissues to radiation and the current widespread use of radiological examinations and treatments, we should be more cautious in the use of these procedures. We should be aware of the risks associated with cerebral interventional procedures and should further optimize the radiation dose for cerebral interventional procedures to reduce the accumulation of RIM risk factors.

## Data availability statement

The original contributions presented in the study are included in the article/supplementary material. Further inquiries can be directed to the corresponding author.

## Ethics statement

Written informed consent was obtained from the individual(s) for the publication of any potentially identifiable images or data included in this article.

## Author contributions

JLi: Investigation, Writing – original draft. XZ: Data curation, Writing – review & editing. CS: Funding acquisition, Writing – review & editing. JLiu: Methodology, Writing – review & editing. JC: Visualization, Writing – review & editing. LY: Formal analysis, Writing – review & editing. YG: Supervision, Writing – review & editing.
